# In vivo analgesic, anti-inflammatory, and sedative activity and a molecular docking study of dinaphthodiospyrol G isolated from *Diospyros lotus*

**DOI:** 10.1186/s12906-020-03030-2

**Published:** 2020-07-25

**Authors:** Abdur Rauf, Tareq Abu-Izneid, Fahad A. Alhumaydhi, Naveed Muhammad, Abdullah S. M. Aljohani, Saima Naz, Saud Bawazeer, Abdul Wadood, Mohammad S. Mubarak

**Affiliations:** 1 Department of Chemistry, University of Swabi Anbar KPK, Swabi, Pakistan; 2 Department of Pharmaceutical Sciences, College of Pharmacy, Al Ain University, Al Ain, UAE; 3 Department of Medical Laboratories, College of Applied Medical Sciences, Qassim University, Buraydah, Saudi Arabia; 4 Department of Pharmacy, Abdul Wali Khan University Mardan, KPK, Mardan, Pakistan; 5 Department of Veterinary Medicine, College of Agriculture and Veterinary Medicine, Qassim University, Buraydah, Saudi Arabia; 6 Department of Woman University, Mardan, Mardan KPK, Mardan, Pakistan; 7 Department of Pharmaceutical Chemistry, Faculty of Pharmacy, Umm Al-Qura University, P.O. Box 42, Makkah, Saudi Arabia; 8 Department of Chemistry, Abdul Wali Khan University Mardan KPK, Mardan, Pakistan; 9 Department of Chemistry, The University of Jordan, Amman, 11942 Jordan

**Keywords:** *Diospyros lotus*, Analgesic, Anti-inflammatory, And muscle relaxation, Molecular docking study

## Abstract

**Background:**

Analgesic, anti-inflammatory, and sedative drugs are available with potential side effects such as peptic ulcer and addiction among other things. In this regard, research is underway to find safe, effective, and economical drugs free of these side effects. In this study, an isolated natural product from ***Diospyros lotus***, was tested for the aforementioned bioactivities.

**Objectives:**

To evaluate analgesic, anti-inflammatory, and sedative potential of ***D. lotus*** extracts in animal paradigms using BALB/c mice as experimental model.

**Methods:**

Analgesic, anti-inflammatory and sedative activities of dinaphthodiospyrol G (**1**) isolated from the chloroform fraction of ***D. lotus*** were evaluated using different experimental procedures. Anti-inflammatory effect was evaluated using the carrageenan and histamine-induced paw edema, whereas the antinociceptive effect was quantified by means of the hot plate analgesiometer. On the other hand, the sedative effect was determined using animal assay for screening the locomotors effects of compound **1**. Compound **1** was also subjected to molecular modeling studies against cyclooxygenase enzymes.

**Results:**

Results from this investigation showed that the extract is devoid of anti-inflammatory and antinociceptive potentials but has a significant sedative effect, whereas the tested compound exhibited 55.23 and 78.34% attenuation in paw edema by carrageenan and histamine assays, respectively. A significant (*p* < 0.001) and dose-dependent antinociceptive and sedative effects were demonstrated by the isolated compound. Molecular docking and dynamics simulation studies of the isolated compound against cyclooxygenase enzyme indicated that compound **1** forms specific interactions with key residues in the active site of the target receptor, which validates the potential use of the isolated compound as cyclooxygenase inhibitor.

**Conclusions:**

Compound **1** exhibited remarkable analgesic, anti-inflammatory, and sedative activities. These findings strongly justify the traditional use of ***D. lotus*** in the treatment of inflammation, pain, and insomnia.

## Background

*Diospyros lotus,* normally known as date plum, lilac persimmon, belongs to family *Ebenaceae*. It is a broadly cultivated species of the Diospyros genus, and is distributed in China, Japan, Asia, and southeast Europe, sub-tropical (Pakistan and India) and tropical regions (America and Africa) [1]. It grows at up to 2000 m elevation; however, it grows best at altitudes below 600 m. Its fruits have an exquisitely rich flavor when completely ripe, and have a astringent taste before they ripen. *D. lotus* has been used in the traditional system of medicine to treat various diseases including febrifuge, promote secretions, as well as a sedative [2]. Different parts of this plant have been documented for multiple medical uses such as leaves as an analgesic, fruits as a febrifuge and sedative, seeds as carminative, and bark as a febrifuge [3]. Likewise, leaf extracts from date plum have been used as anti-tobacco toffees. Traditionally, *Diospyros* species are used as a therapeutic medicine for the treatment of hiccups, bedwetting, insomnia, hypertension, dyspnea, pains (muscular and joints), intestinal worms, and fever [4, 5]. Isolated phytochemicals and various extracts from different parts of ***D. lotus*** have also been reported to possess strong anti-proliferative potentials [6]. Photochemistry of ***D. lotus*** documented various classes of secondary metabolites such as terpenes, steroids, phenolic compounds, and dimeric naphthoquinones. Research findings indicated that quinone molecules are the main moieties in currently available drugs which are used for the treatment of cancers [7].

Additionally, few naphthoquinones (plumbagin) isolated from plant matrices of various *Diospyros* species have displayed significant potent cytotoxic characteristics [8]. Naphthoquinones are bioactive phytochemicals isolated from several families of higher plants and possess redox properties in addition to being involved in numerous biological oxidative processes [9]. Moreover, naphthoquinones are considered fundamental structures in the field of medicinal chemistry due to their biological potency. They are associated with the electron transport chain in the metabolic pathways, mostly in numerous biological oxidative processes [9]. Among the secondary metabolites, quinones are mainly involved in reduction processes and can act as oxidizing agents. In folk medicine, medicinal plants containing naphthoquinones have been used to treat various diseases such as cancer, inflammation, and as sedative. Naphthoquinone’s derivatives exhibited significant analgesic, antioxidant, antipyretic, and anti-inflammatory activities [10–12]. Similarly, naphthoquinone’s class has been reported with antihistaminic effect [13]. This antihistaminic type action might be similar to H1 receptor blocker (pheniramine), which is sedative [14]. On the basis of the preceding discussion, this study was designed to investigate the analgesic, anti-inflammatory, and sedative potential of extract and compound **1** isolated from ***D. lotus*** roots.

## Methods

### Plant material

Roots of ***D. lotus*** were obtained in May 2009 from Razagram Distt; Dir Khyber Pakhtunkhwa Pakistan. The plant specimen was identified by Prof. Dr. Abdur Rashid, a well-known taxonomist, Department of Botany, University of Peshawar, KPK, Pakistan. A voucher specimen no [Bot. 20,036 (PUP)] was deposited at the herbarium located at the said Department.

### Extraction and isolation

**Fig. 1:**
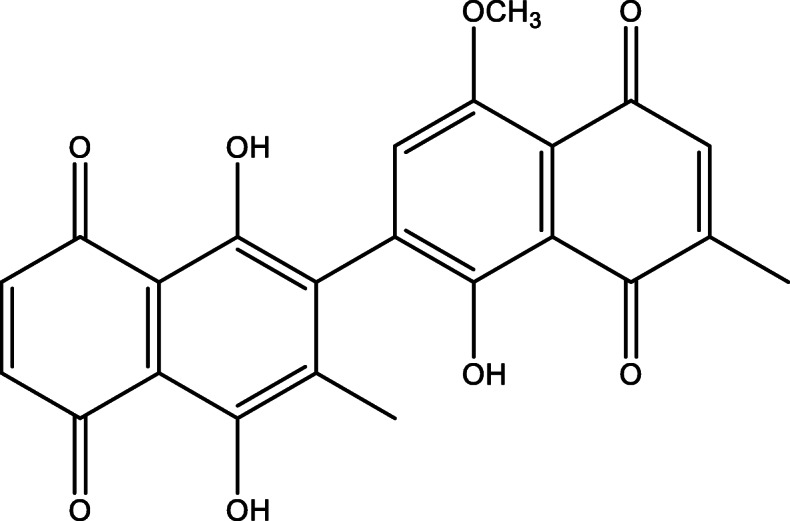
Chemical structure of dinaphthodiospyrol G isolated from *D. lots*

Shade dried roots (13.88 kg) were powdered by using a local grinder machine. The ground plant sample was subjected to cold extraction using chloroform for 16 days. The obtained extract was concentrated at low temperature and pressure to afford a combined crude extract (198 g). The chloroform extract (198 g) was subjected to soxhlet extraction to remove dyes and fatty acids, and resulted in 166 g; the extraction process was carried out according to published procedures [15]. This step was followed by subjecting 30 g of the chloroform fraction to normal phase column chromatography (CC) using silica gel-60 (Merck-5 × 60 cm). The column was eluted with *n*-hexane/ethyl acetate (100,0 to 0:100) solvent system which yielded 10 sub-fractions (SM-1 to SM-10). Based on the TLC profile, sub-fraction SM-8 (1.98 g) was further subjected to repeated normal phase column chromatography (CC) by eluting the column with *n*-hexane/ethyl acetate solvent system (87,13), to afford compound **1** (Fig. 1). Compound **1** was identified as 5,8,4-trihydroxy-1-methoxy-6,6-dimethyl-7,3-binaphtyl-1,4,5,8- tetraone using spectroscopic techniques, such as 1D and 2D NMR [3].

### Animals

Healthy BALB/c mice (25–30 g) acquired from the National Institute of Health (NIH) Islamabad-Pakistan were used throughout this investigation. Animals were given free access to standard diet and water, and were kept under standard laboratory conditions mentioned in the animal by laws approved by the ethical committee (SOU/Pharm-22) of Pharmacy Department, University of Swabi, Swabi, Pakistan. After the experimental procedure, animals were sacrificed by cervical dislocation and disposed properly. Animals were randomly divided into various groups with six animals each (*n* = 6), including negative control treated with normal saline, positive control and tested groups treated with our test sample.

### Anti-inflammatory screening

Anti-inflammatory effect of the chloroform extract and compound **1** was assessed using the carrageenan and histamine-induced paw edema according to a published procedure [16]. In brief, animals were arbitrarily divided into several groups of six animals each, including negative control (10 mL/kg saline) and positive control (Diclofenac, 5 mg/kg/, Loraditine 2 mg/kg)). Tested groups were treated with chloroform extract (50, 100, and 200 mg/kg) and compound **1** (0.5, 5, 10, and 15 mg/kg). Thirty minutes after the intraperitoneal treatments, 1% carrageenan (0.05 mL) or 0.1 mL of histamine solution (0.5%) was subcutaneously injected in the right hind paw (sub plantar tissues) of each mouse. Inflammation in each mouse was recorded after 1, 2, 3, 4, 5, and 6 h of carrageenan /histamine administration through a plethysmometer (LE 7500 plan lab S.L). Anti-inflammatory activity (percent inhibition) against paw edema was obtained through the following formula:

$$ \mathrm{Inhibition}\ \left(\%\right)=\left(\mathrm{A}-\mathrm{B}/\mathrm{A}\right)\times 100 $$

$$ \mathrm{Where},\mathrm{A}=\mathrm{paw}\ \mathrm{edema}\ \mathrm{of}\ \mathrm{negative}\ \mathrm{control},\mathrm{and}\ \mathrm{B}=\mathrm{paw}\ \mathrm{edema}\ \mathrm{of}\ \mathrm{tested}\ \mathrm{group} $$

### Hot plate assay

In this test, animals were starved for 3 h before the start of the test and were distributed in different groups of six animals each. Group I was administrated with normal saline (10 mL/kg) as control for statistical analysis, group II was administered with tramadol (5 mg/kg), and the rest of the groups were administered with the chloroform extract (50, 100, and 200 mg/kg) and isolated compound (2.5, 5, 10, and 15 mg/kg). Hot plate analgesiometer (Havard apparatus) was used for the determination of central antinociceptive effect following our published protocols [16]. A cut off time (25 s) was used for each animal to avoid tissue damage. After 30 min of sample administration, each animal was tested for analgesic effect on the hot plate, and the latency time was recorded in seconds. Latency was recorded again after 60, 90, and 120 min, and the percent effect was determined following our published method [16].

### Sedative activity

Animals were grouped as above except for positive control where they were treated with diazepam 0.5 mg/kg. The open field apparatus experiment was performed as per reported methods outlined by Muhammad et al. [16]. Animals were classified in different groups. The positive control group was treated with diazepam and negative control group was injected with saline. The tested groups were treated with extract and isolated compound with different doses. According to this procedure, after 30 min of treatment, each animal was placed in a special box and the lines crossed by each mouse were counted. The animal with hindered movement was considered to be sedated and animals that crossed more lines were considered to be nonsedated.

### Molecular dynamics simulation

Molecular docking study of compound **1** was conducted against the anti-inflammatory target, cyclooxygenase receptor-1 (COX-1). The crystal structure of the targeted receptor was retrieved using PDB code 4O1Z from protein databank (www.rcsb.org). The 3D structure of the isolated compound was built in the molecular operating environment (MOE) using MOE-builder module (Chemical Computing Group. Inc). The structural coordinates of the target was subjected to 3D protonation and energy minimization up to 0.05 Gradient using MMFF94s force field implemented in the MOE software. Next, protonation was performed using default parameters in MOE and was energy minimized to get a minimum energy conformation of the target. Finally, optimized structures were used for molecular docking purposes, utilizing the default molecular docking standard protocol in MOE. Assessment was chosen for the top-ranking docked complex based on the docking score (S) and the protein-ligand interaction (PLI) profile. The docked complex was used to performing molecular dynamics simulation to evaluate the stability of the complex by AMBER18 software package (University of California, San Francisco. Inc). The dinaphthodiospyrol G isolated compound from *D. lots* was parameterized using the generalized Amber force field (GAFF) [17]. Antechamber module was used for assigning the GAFF atom type [18]. MD simulations and analysis were conducted in AMBER version 2018 [19]. The LEaP module was used to add hydrogen atoms to the complex. Next, we have added the counter ions to preserve the system neutrality. The system was solvated in an octahedral box of the TIP3P water model with a cut-off 10 Å buffer. The Particle Mesh Ewald method has been used to evaluate the distant intermolecular interactions [19]. The ff14SB force field was used for conducting the MD simulation [123], and the SHAKE Algorithm was applied to constrain all covalent bonds involving hydrogen atoms with a tolerance of 10 Å to 5 Å [20, 21]. We used the CUDA version of PMEMD to accelerate the MD simulation process. The steepest descent method was used to minimize the solvated system for 20, 000 steps, then 400 ps heating, and 200 ps equilibration in the NVT ensemble. The temperature and pressure were combined with a constant time of 01 ps. Finally, the Langevin’s algorithm was used for the isotropic position scaling, and a relaxation with a time of 2.0 ps [22].-.

### Statistical analysis

All obtained results of this screening are presented as the mean ± standard error of the mean (SEM) to find the significant difference (*p* < 0.05 or 0.01) amongst experimented groups. Data were subjected to one-way analysis of variance (ANOVA). Statistical analysis was performed with the aid of Dunnett’s multiple assessment tests using GraghPad prsim 5; differences were considered significant at *p* ≤ 0.05.

## Results

### Anti-inflammatory effect

**Fig. 2:**
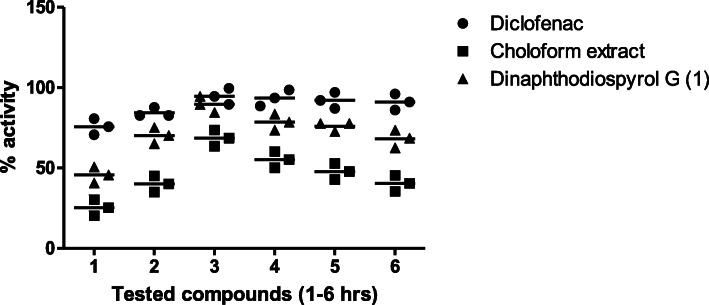
Anti-inflammatory effect of chloroform extract (200 mg/kg) and Dinaphthodiospyrol G (15 mg/kg) isolated from *D. lotus* on carrageenan paw in mice. All values are represented as mean ± S.E.M for all groups (*n* = 6)

Our findings revealed that the extract exhibits a significant impact at a higher dose in the carrageenan-induced paw edema. At the early inflammatory phase, 25% attenuation in edema was observed which was improved with time. The maximum reduction (55.23%) was noticed after the 4th h of experimental duration. On the other hand, the anti-inflammatory effect of the tested compound was 45.65% in the initial phase and 89.56% in the secondary phase, as shown in Fig. 2.

**Fig. 3:**
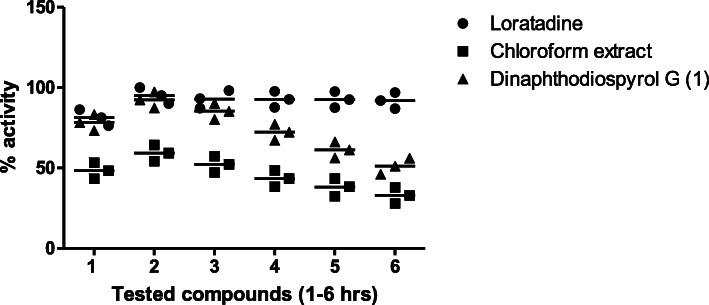
Anti-inflammatory effect of chloroform extract (200 mg/kg) and Dinaphthodiospyrol G (15 mg/kg) isolated from *D. lotus* on Histamine paw edema in mice. All values are represented as mean ± S.E.M for all groups (*n* = 6)

In the histamine-induced paw edema, the extract exerted a reduction in paw edema from the initial phase (48.34%, at the first hour) and was maintained up to the secondary phase (52.34%, at the 3rd hour). A significant (78.34%) anti-inflammatory effect was observed against compound **1** from the 1st hour of the experiment and remained for the 3rd hour, as shown in Fig. 3.

### Antinociceptive effect

A significant (*p* < 0.001) central antinociceptive effect was noticed at various doses as presented in Table 1. Compound **1** increased the latency time from the start of the experiment and maintained a significant increase (*p* < 0.001) up to 120 min. No significant effect was noticed against lower tested doses. The extract was devoid of any pain-relieving potential.

**Table 1:** Analgesic activity of Dinaphthodiospyrol G isolated from *D. lots*

Groups	(Doses mg/kg)	Time (Minutes)			
		30	60	90	120
Saline	10 mL/kg	9.21 ± 0.09	9.19 ± 0.08	9.20 ± 0.011	9.18 ± 0.12
Tramadol	10 mL/kg	24.61 ± 0.08***	26.99 ± 0.98***	25.60 ± 0.88***	25.00 ± 0.42***
Chloroform extract	50	8.60 ± 0.33	8.90 ± 0.45	8.98 ± 0.64	8.19 ± 0.90
	100	9.00 ± 0.54	9.55 ± 0.52	9.70 ± 0.70	8.98 ± 0.80
	200	10. 05 ± 0.20	10.35 ± 0.50	10.25 ± 00.55	10.00 ± 0.40
Dinaphthodiospyrol G	2.5	16.11 ± 0.44	17.38 ± 0.67*	16.98 ± 80	16.77 ± 1.02
	5	17.84 ± 0.54*	19.01 ± 0.70**	18.50 ± 0.88*	18.13 ± 1.06*
	10	19.50 ± 0.60**	20.97 ± 0.78**	20.00 ± 0.80**	19.99 ± 1.12**
	15	21.50 ± 0.77***	22.98 ± 0.81***	22.08 ± 0.95***	21.80 ± 1.21***

Data expressed as the mean ± standard error of the mean (S.E.M.) of latency time in seconds for each group (*n* = 6), statistically, analysis of variance (one way) was done followed by multiple comparison through Dunnett’s post-hoc test; Where, *p* < 0.05 = *; *p* < 0.01 = **; *p* < 0.001 = ***.

### Sedative effect

Shown in Table 2 are results related to the sedative effectof extract and of compound **1**. The extract failed to induce sedation at any measured dose, whereas compound **1** demonstrated a significant (*p* < 0.001) sedative effect as shown by hindering the mobility of animals in a special box.

**Table 2:** Sedative activity of dinaphthodiospyrol G isolated from *D. lots*

Treatment	Doses (mg/kg)	Line crossed
Diazepam	0.5	6.86 ± 0.11***
Chloroform extract	50	104.54 ± 2.00
	100	99.60 ± 2.34
	200	96.20 ± 2.70
Dinaphthodiospyrol G	2.5	88.98 ± 1.86*
	5	74.00 ± 1.58**
	10	62.98 ± 1.32**
	15	51.98 ± 1.20***

Data expressed as the mean ± standard error of the mean (S.E.M.) of number of lines crossed for each group (*n* = 6), statistically, analysis of variance (one way) was done followed by multiple comparison through Dunnett’s post-hoc test; Where, *p* < 0.05 = *; *p* < 0.01 = **; *p* < 0.001 = ***.

### Molecular dynamics simulation

Docking results revealed that the isolated compound shows a fit-well pattern of binding in the active site of the receptor. The protein-ligand interaction profile (PLI) showed that the compound adopted both hydrogen donor (H-donor) and acceptor (H-acceptor) with various binding energies (kcal/mol). Further, the molecular dynamics simulation results of the docked complex revealed that compound **1** shows a very stable behavior throughout a 50 ns MD simulation time.

## Discussion

***D. lotus*** is one of the indigenous medicinal plants which is traditionally used as sleep inducer, analgesic, and antipyretic. The crude extract of this plant has been reported with significant anti-inflammatory, sedative, and anti-nociceptive effect [23]. In addition to these effects, researchers reported on the effect of ***D. lotus*** as antioxidant [23], antipyretic [19], and anti-HIV [24]. The crude extract of any plant contains constitutes responsible for biological activities. Therefore, isolation of these constituents followed by screening for biological actions is essential to find the extracted chemical constitute responsible for any biological activity. In the current study, the isolated compound from ***D. lotus*** has been tested for anti-inflammatory, anti-nociceptive, and sedative effect.

Naphthaquinone derivatives with significant analgesic, antipyretic, and anti-inflammatory effect [10, 11, 25, 26], have been reported. With this in mind, we tested compound **1** for anti-inflammatory effect. The tested compound attenuated inflammation in both models, i.e. carrageenan and histamine-induced paw edema. Carrageenan induces inflammation in two phases [11, 12], the initial phase edema is attributed to the local release of histamine, bradykinins, and serotonin while the other phase is due to overproduction of prostaglandins (PG). Our findings indicate that the chloroform fraction was devoid of any anti-inflammatory potential which might be due to agonist and antagonist chemical constitutes in plants extracts [27]. The extract failed to resist the edema in all tested doses. However, compound **1** inhibited the biphasic edema induced by carrageenan, which is an indication that this isolated molecule is a histamine and PG blocker.

Similarly, naphthaquinones have been reported with antihistaminic effect [28–31]. This antihistaminic type action might be similar to H_1_ receptor blocker (pheniramine), which is sedative [13] because, in the current work, our tested compound is a significant sedative. PG is the product of arachidonic acid by cyclooxygenase (COX). The majority of COX inhibitors are painkiller, antipyretic, and anti-inflammatory with a potential side-effect to the stomach. Therefore, the discovery of new, safe, and effective COX inhibitor is a big challenge to researchers. In the thermal induced pain paradigm, no antinociceptive effect was revealed by the extract, but a significant effect was observed for the tested compound.

Docking results revealed several critical interactions including H-donor and H-acceptor with key residues which might have a role in the enhancement of the corresponding activity.

**Fig. 4:**
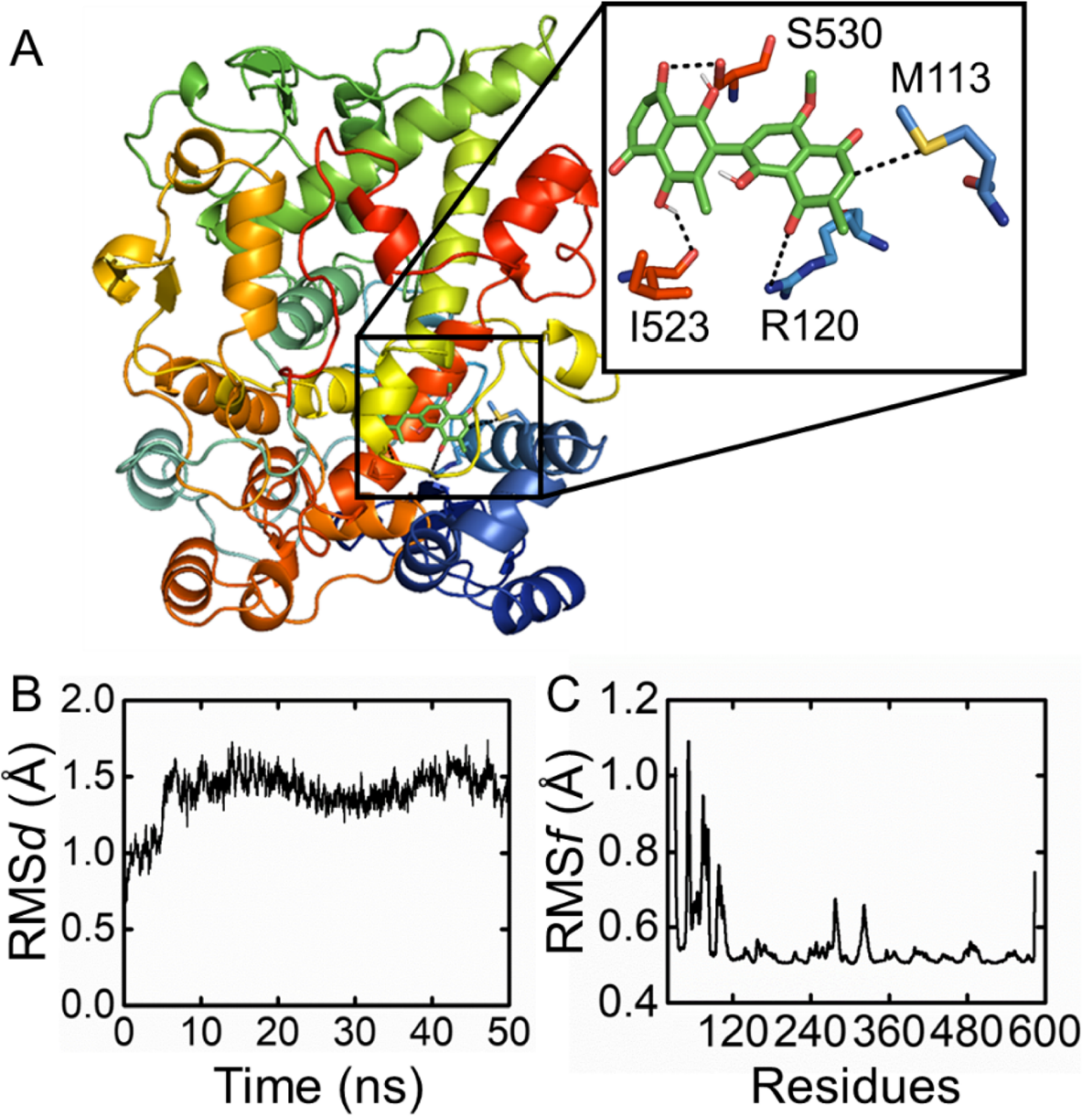
**a** The protein-ligand interaction pose of the docked complex. **b** Indicate the RMS*d* graph for the isolated compound, while **c** for RMS*f* graph

Details of the critical interactive residues are shown in Table 3 and Fig. 4a. A total of 50 ns MD simulations were performed to check the stability of the complex in an explicit water environment. The root-mean-square-deviation (RMS*d*) was calculated to determine the stability validation of the complex, which was a key parameter for evaluating the equilibrium of the MD trajectory for the primary chain atoms of the protein system along with the MD simulation. Results indicated that the RMS*d* value of the isolated compound increases (12 ns) and suffers from high fluctuation but becomes stable after 17 ns. The gradual increase in RMS*d* of the complex might be due to grasping the stable states where first they attained the exited state and then oscillated afterward. The average coordinate was extracted from a total of 50 ns MD simulations and showed that the compound is stable with adopted several key interactions with active site residues. The following analysis was obtained from the 50 ns trajectory calculations, which show that the stable behavior of the isolated compound strongly inhibited the target and bonded explicitly to the binding site (Fig. 4b).

**Table 3:** The protein-ligand interaction (PLI) profile for an isolated compound

Ligand	Receptor	Interaction	Distance	E (kcal/mol)	Docking Score
C 19	SD MET 113	H-donor	3.73	−0.8	−8.83694744
O 22	O ILE 523	H-donor	3.24	−1.3	
O 24	OG SER 530	H-acceptor	2.81	−0.7	
O 29	NH2 ARG 120	H-acceptor	3.71	−1.1

We analyzed the fluctuations of residues in terms of root-mean-square fluctuation (RMS*f*) to determine how compound **1** affected the side chain of the target receptor residues. RMSf results revealed a high fluctuation in the binding site residue where compound **1** adopted favorable interactions with critical residues, while other regions showed local fluctuation (Fig. 4c). These results delineate that compound **1** showed a very stable behavior against the target receptor and gives us a clue regarding the best potential of this compound against the targeted receptor. The significant anti-inflammatory, anti-nociceptive, and sedative effects of compound **1** provide a strong scientific justification to the traditional uses of ***D. lotus*** in the treatment of inflammation, pain, and insomnia.

## Conclusions

***D. lotus*** is traditionally used as painkiller, anti-inflammatory, and sedative. Results from this investigation indicated that 5,8,4-trihydroxy-1-methoxy-6,6-dimethyl-7,3-binaphtyl-1,4,5,8- tetraone (**1**) from the chloroform fraction exhibits significant anti-inflammatory, anti-nociceptive, and sedative effects. Thus our findings provided scientific rational to the traditional use of ***D. lotus*** for the treatment of various diseases. Additionally, compound **1** is a novel candidate for further detailed screening to ascertain its clinical use. However, more detailed studies are required to establish the safety and efficacy of compound **1** and the extract.

## Data Availability

The data associated to this study are available from corresponding author.

## Abbreviations

CC: Column Chromatography
1D: One-Dimensional
2D: Two-Dimensional
3D: Three-Dimensional
MOE: Molecular Operating Environment
PLI: Protein-Ligand Interaction
PG: Prostaglandins
COX: Cyclooxygenase
GABA: Gamma-Aminobutyric Acid
RMSd: Root-Mean-Square-Deviation
MD: Molecular Docking
RMSf: Root-Mean-Square Fluctuation
